# Pharmacological and Ethnomedicinal Overview of *Heritiera fomes*: Future Prospects

**DOI:** 10.1155/2014/938543

**Published:** 2014-07-21

**Authors:** Imran Mahmud, Md Khirul Islam, Sanjib Saha, Apurba Kumar Barman, Md Mustafizur Rahman, Md Anisuzzman, Taufiq Rahman, Abdullah Al-Nahain, Rownak Jahan, Mohammed Rahmatullah

**Affiliations:** ^1^Pharmacy Discipline, Life Science School, Khulna University, Khulna 9208, Bangladesh; ^2^Department of Pharmacology, University of Cambridge, Tennis Court Road, Cambridge CB2 1PD, UK; ^3^Department of Pharmacy, University of Development Alternative, Dhanmondi, Dhaka 1209, Bangladesh; ^4^Department of Biotechnology & Genetic Engineering, University of Development Alternative, Dhanmondi, Dhaka 1209, Bangladesh

## Abstract

Mangrove plants are specialized woody plants growing in the swamps of tidal-coastal areas and river deltas of tropical and subtropical parts of the world. They have been utilized for medicinal and other purposes by the coastal people over the years. *Heritiera fomes* Buch. Ham. (family: Sterculiaceae) commonly known as Sundari (Bengali) is a preeminent mangrove plant occurring in the Sundarbans forest located in the southern part of Bangladesh and adjoining West Bengal province of India. The plant has applications in traditional folk medicine as evidenced by its extensive use for treating diabetes, hepatic disorders, gastrointestinal disorders, goiter, and skin diseases by the local people and traditional health practitioners. A number of investigations indicated that the plant possesses significant antioxidant, antinociceptive, antihyperglycemic, antimicrobial, and anticancer activities. Phytochemical analyses have revealed the presence of important chemical constituents like saponins, alkaloids, glycosides, tannins, steroids, flavonoids, gums, phytosterols, and reducing sugars. The present study is aimed at compiling information on phytochemical, biological, pharmacological, and ethnobotanical properties of this important medicinal plant, with a view to critically assess the legitimacy of the use of this plant in the aforementioned disorders as well as providing directions for further research.

## 1. Introduction

Finding healing power in plants is an ancient idea. Man has always been in search for agents to cure various ailments. Since antiquity, medicinal plants and herbs have been in use for the eradication of diseases and human sufferings. According to some estimates, almost 80% of the present day medicines are directly or indirectly obtained from medicinal plants [1]. In developing countries, medicinal plants constitute a precious natural wealth and contribute a great deal to its health care programs. They play an important role in ensuring primary health care facilities and services to countryside people. They also serve as important healing agents as well as vital raw materials for the preparation of conventional and modern medicines.

Mangrove forest contains specialized plant species that grow up at the edge between sea and land in subtropical and tropical regions of the world where they exist in high temperature, strong winds, extreme tides, high salinity, and anaerobic soil. No other group of plant species exists in this universe with such physiological and morphological adaptations to severe conditions. The unique ecology, morphological characteristics, and traditional uses of mangrove plants have drawn the attention of researchers over the years. Mangroves are distributed in 112 countries and territories. Global coverage of mangroves is almost 18 million hectares [2]. They are biochemically unique in nature and considered as a source of novel natural products. Usually mangroves are rich in polyphenols and tannins [3]. Mangrove leaves contain phenols and flavonoids that serve as ultraviolet (UV) screen compounds. Substances in mangroves have long been used in folk medicines to treat diseases [4]. Extracts of various parts of mangrove plants have significant activity against animal, human, and plant viruses including human immunodeficiency virus [5].

Mangrove plant species are atypical from common terrestrial plants in that they can tolerate high salt concentration and remain submerged in saline water. Because of the scant distribution of the mangrove forests, mangrove plant species are still almost unacquainted to a vast population. Ancient people used mangrove plant species scarcely because they could hardly enter these areas [6]. However, at present a vast population lives beside these forested areas and they are heavily dependent on the mangrove forests to earn their livelihood as well as for healthcare. These areas are quite distinct from the cities and there are hardly any hospitals or doctors for their treatment. They are totally dependent on local traditional health practitioners (THPs) for eradicating various ailments and sufferings. Mangrove plant species are used extensively for their primary healthcare by the THPs and the rural people living in these areas. The comprehensive uses of various mangrove plant species in human sufferings provide strong evidence of their healing power and demands further research. But there is lack of sufficient data on mangrove plant species due to the scarcity of these species as well as difficulty in collection and identification. Moreover, adequate information about their ethnomedicinal uses is not available.

**Figure 1:**
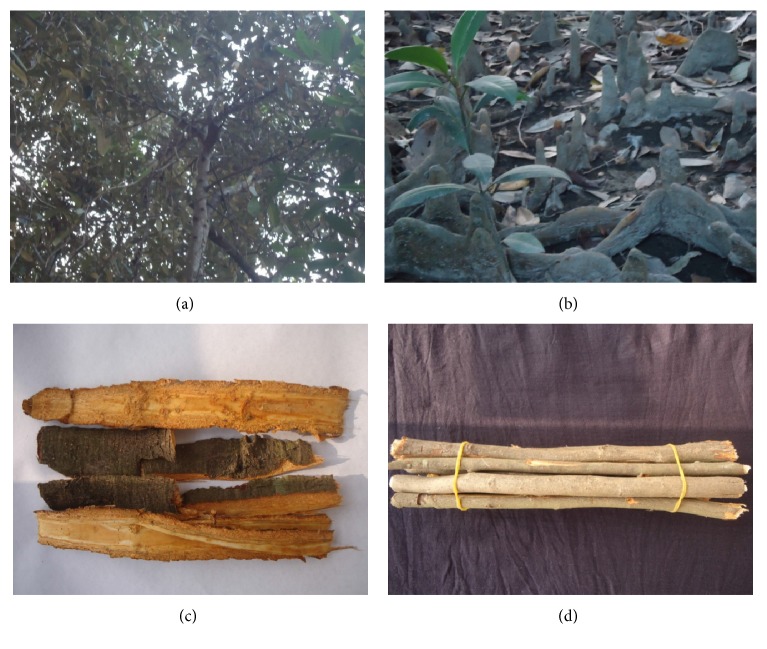
Various parts of* Heritiera fomes*. Clockwise from top left: tree, pneumatophores, twigs, and barks.

**Figure 2:**
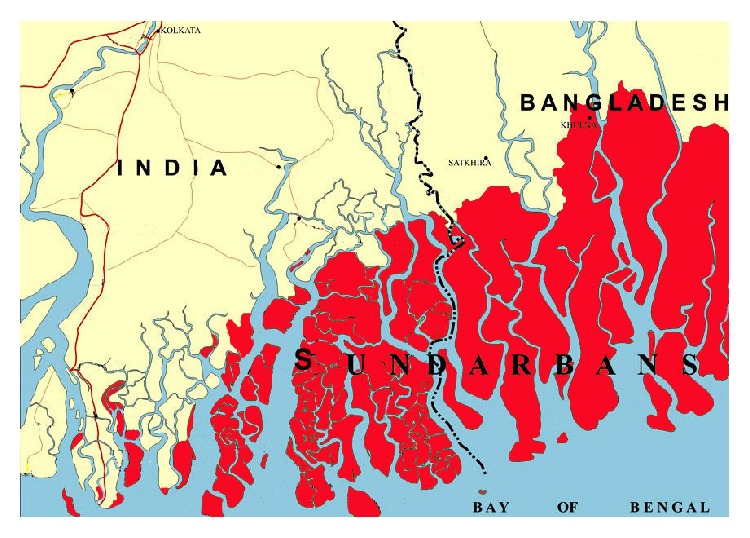
Map of the Sundarbans.

*Heritiera fomes* Buch. Ham. (Syn.:* Heritiera minor* or* Amygdalus minor*) (Figure 1) is an evergreen moderate size tree growing abundantly in Sundarbans [7]. The trees attain up to 25 m in height; trunk is about 50 cm in diameter at the base is prominently buttressed. The young branches of the trees are covered with shining golden-brown scales.* H. fomes* is an important mangrove species having ethnomedicinal uses in traditional medicines. The people living beside the Sundarbans (Figure 2) use this plant extensively for treating various ailments. It is used in gastrointestinal disorders including diarrhea, dysentery, constipation, indigestion, and stomachache. It is also recommended for skin diseases including dermatitis, rash, eczema, boils, itch, scabies, sores, infections, and hepatic disorders including jaundice, hepatitis. It is also useful for treating diabetes and goiter. It is a good insect repellent [8] and has wound healing activity [9].

*H. fomes* possesses significant antioxidant [10], antinociceptive, antihyperglycemic [11], antimicrobial [12], and anticancer activities [13]. It is also useful in cardiovascular diseases [14]. Moreover the plant is widely used by the coastal people for different purposes. The wood of the plant is used to make boats, making poles, and construction purpose [15].

It is relevant in this context to discuss also ethnomedicinal and pharmacological reports on other mangrove species, especially belonging to the* Heritiera* genus.* Heritiera utilis* is an evergreen tree. Seed oil obtained from seeds is reported as edible and used as aphrodisiac, while ground seeds are used to abscesses as a poultice. Bark decoction of* H. utilis* is used to skin affections occurred by leprosy and given orally as an aphrodisiac [16].

Seed extracts of* H. littoralis* are applied for the treatment of dysentery and diarrhea [17]. Traditionally, the leaves and stems also have been utilized against dysentery and diarrhea. The sap is proven to be various types of poison including fish poison, arrowhead, and spearhead poison [17, 18]. The leaves and seeds of this plant are taken as edible in the Nicobar and Andaman islands [19]. Sometimes, twig is used as chew sticks as well as tooth brushes [20].

This review aims to compile the pharmacological activities, ethnomedicinal, and phytochemical reports of* H. fomes*.

## 2. Botanical Features

### 2.1. Habitat

The plant is found abundantly in Sundarbans, which is the world's largest mangrove forest located at the southern part of Bangladesh and Indian state of West Bengal. The forest encompasses a land area of 6017 sq·km of which 1874 sq·km is covered by river areas. The forest is surrounded by the Bay of Bengal in the south, while polders and agricultural lands border the forest in the north. The forest is flush and the maximum ground elevation is 3 meter above the mean sea level.

Through the subsidence and down wrapping of sediments, the land of the forest has developed. Sediments are being deposited to the soil through the flowing of river and sea water. The forest has interesting ecological characteristics with a temperature ranging from 20.4°C to 31.5°C. The annual rainfall is from 1640 to 2000 mm. As the land is on sea interface, the mangrove species are always associated with saline seawater. Unlike other mangrove species* Heritiera fomes* prefers extremely low saline condition (5–15 psu) [21]. There is a popular credence that the “Sundarbans” derived its name from the Sundari (*Heritiera fomes*) trees, a major component of the forest. It covers 52.7 percent of the area and constitutes about 63.8 percent of the standing volume [22].

### 2.2. Morphology

It is an evergreen medium sized tree, growing up to 25 m in height. The leaves are dark green and have short petioles of about 1 cm. They are grouped toward the ends of the branches. At the age of three years, the species begin to produce pneumatophores. The height of the pneumatophores is about 50 cm.* H. fomes* is the only* Heritiera* species which produces pneumatophores. Pneumatophores are one type of excessively branched roots that are negatively geotropic and come out of the mud surface to access the atmospheric oxygen. The sapwood is pinkish grey and heartwood dark to radish dark brown. Wood is hard, heavy, and durable. The flowers are unisexual and incorporated in panicles. They consist of 5 stamens fused to form cylinder dumbbell known as pistilloid. Usually the species flowers in March and April. Fruits are light green and become brown with ripening. They are single seeded with fleshy endosperm. Seed size varies between 3–5.5 cm long and 3.5–5 cm wide. Seed shedding occurs during June and July [23].

Another mangrove species named* Heritiera littoralis* possesses some similar morphological characteristics to* H. fomes*.* H. littoralis* (Sterculiaceae) is a tree attaining up to 25–30 feet. Leaves are dark green and occur 10 to 23 cm × 4 to 10 cm broadly elliptic with acute apex having cuneate base. Base may also be rounded or entirely covered beneath with minute silvery hairs. Petioles are brown coloured and become 1.1 to 2.5 cm long. Bark is longitudinally furrowed and young parts covered with stellate hairs. Long buttresses are seen on soil at the bottom of the bole. Flowers are small, much branched, unisexual in tomentose, druping, and axillary panicles in the upper axils. Male flowers are slightly smaller than female flowers. The species flowers from June to August. Fruits are one seeded, 6 to 9 cm long, and 5 to 6 cm wide. Mature fruits are collected during January to April [24].

## 3. Ethnomedicinal Reports

Ethnomedicine (sometimes synonym for traditional medicine) is known as a subclass of medical anthropology (study of human disease and health care systems), which not only concern with the relevant written documents but also those people whose practice, experience, and knowledge have been verbally transmitted to next generation over the future centuries. Drug discovery and anthropological research are carried out based on scientific ethnomedical studies.

The mangrove forest in Bangladesh is home to various species of fauna and flora and is economically valuable due to supplement of medicinal support besides providing wood and other resources like honey, crabs, and fish.* H. fomes* has a restricted distribution in the Sundarbans, Bangladesh. This species is a well-known mangrove plant for its significant traditional use(s) by the local traditional health practitioners (THPs) against various diseases in the southern areas of Bangladesh.* H. fomes* leaves, roots, and stems are used by rural people for the treatment of gastrointestinal disorders, skin diseases, and hepatic disorders [13, 25]. Bark is taken for diabetes and goiter in rural areas [11]. This plant is also used as herbal medicine to cure pain and fever locally (Table 1). People settling in these regions are out of reach of modern medicine.

According to the IUCN Red List Categories and Criteria, this plant is facing danger and quickly disappearing in many regions because of coastal development. Due to excess-harvesting, diversions of water in Ganges Basin, and salinity fluctuations, this tree is threatened or is facing extinction [33]. This review has been done in attempt towards preservation of the ethnobotanical and ethnomedicinal knowledge and in order to protect and document the biodiversity of the Sundarbans.

## 4. Phytochemical Constituents

**Figure 3:**
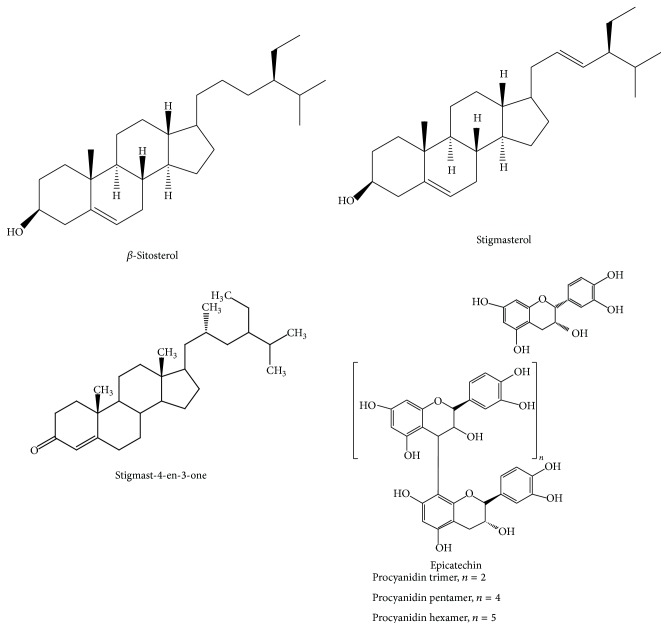
Structures of some phytochemical constituents reported from* H. fomes*.

*Heritiera fomes* is a promising mangrove species. In spite of having enormous potential, few reports are available on this species about its biological activities and the active principles accountable for such activities (Table 2).* H. fomes* contains 0.25% chlorophyll a, 0.09% chlorophyll b, 0.11% carotenoids, 39.45% polyphenols, 21.12% tannins, and titratable acid number (TAN) is 34.50 [30]. The presence of reducing sugars, saponins, alkaloids, glycosides, tannins, steroids, flavonoids, and gums has been demonstrated by phytochemical exploration of leaves extract [25]. Leaf contains 29.22% protein [30]. The bark of* H. fomes* contains 7–36% tannin [31] and a high content of proanthocyanidins [32]. According to their structures, tannins are distributed into two groups such as water soluble tannins (hydrolysable) and proanthocyanidins (condensed tannins) [34, 35]. Stem bark contains high amount of procyanidins. Trimeric, pentameric, and hexameric procyanidins have been identified from the plant. *β*-Sitosterol, stigmasterol, and stigmast-4-en-3-one (Figure 3) were also found from the NMR spectroscopy of CHCl_3_ extract of the* H. fomes* [12].

## 5. Pharmacological Activities of* Heritiera fomes* and Comparison with Other Mangrove Species

A literature survey showed that all plant parts of* Heritiera fomes* are used in the treatment of different ailments; leaves and seeds are reported for the gastrointestinal disorders (diarrhea, dysentery, constipation, acidity, indigestion, and stomachache) [8, 9, 26]. The bark and stem bark are well-reputed remedies for diabetes and skin diseases (dermatitis, eczema, boils, abscess, acne, sores, and rash) [8, 11, 26]. Local people are seen to use twig to clean teeth and relive cough [29].

*Heritiera fomes* possesses significant groups of phytochemical constituents that have been described in other plants to have a wide range of pharmacological activities. Plant saponins reportedly have some important biological activities such as spermicidal [36], molluscicidal [37], antimicrobial, anti-inflammatory, and cytotoxic activities [38, 39]. It has been established that flavonoids reportedly possess antioxidant activity toward a wide variety of oxidizable compound(s) [40]. Several* in vitro* studies established that flavonoids are able to scavenge various radicals such as hydroxyl, peroxyl, and superoxide radicals and thus block various steps in the arachidonate cascade via lipoxygenase cyclooxygenase-2 [41]. The presence of polyphenols in plant species may be responsible for the prevention of diseases. Actually polyphenols are one of the key constituents of the defense mechanism generated by medicinal plants [42]. They reportedly act as free radical scavengers, antimicrobial, and anticancer agents [43, 44]. Polyphenolic compounds also have the ability to inhibit human platelet aggregation [45].

Various biological activities such as antibacterial, antiherpetic, cytotoxic, antineoplastic, and anthelmintic are exhibited due to the presence of tannins and proanthocyanidins in plants, and they provide defense against herbivores and invading parasites [35, 46]. Proanthocyanidins are flavonoid polymers and supposed to have potential against diarrheal diseases [47, 48]. A number of studies have shown the efficacy of some flavonoids as antidiarrheal agents such as catechins, proanthocyanidins, and proanthocyanidin-rich extracts [49, 50]. They are good antioxidants and can inhibit dysentery caused by* Entamoeba histolytica* lectin [51] and* Shigella dysenteriae* toxin [52]. The above discussion provides the rationalization of the ethnomedicinal uses of this plant in various gastrointestinal disorders. So, this plant might be a potential source of antidiarrheal phytomedicines. Free radicals are detrimental because they can take part in various reactions adverse to the body due to their side chain properties. Many life processes produce free radicals [53], and as such, scavenging or reducing the amount of free radicals in the body through phytochemicals can lead to a healthier body system.

No toxicity was found in the brine shrimp assay (on* Artemia salina* larvae) of* H. fomes* extracts (10–1000 *μ*g/mL) [12]. Several* in vitro* studies reveal that the plant possesses significant antidiabetic, antimicrobial, antioxidant, antinociceptive, and anticancer potentials (Table 3).

### 5.1. Antidiabetic Activity

The experimental study reported that bark extracts of* Heritiera fomes* are effective in mice at the dose of 250 and 500 mg/kg body wt. The extracts (at a dose of 250 mg/kg body weight) reduced the level of serum glucose up to 49.2% at 60 min in Swiss albino mice following glucose loading, while a standard, glibenclamide (at a dose of 10 mg/kg body weight) reduced serum glucose by 43.5%. At the dose of 250 and 500 mg/kg body wt, the extracts of* Heritiera fomes* bark reduced serum glucose levels by 35.6 and 44.7%, respectively, following 120 min of glucose loading, while glibenclamide (at a dose of 10 mg/kg body weight) reduced serum glucose level by 30.1% [11].

Daily oral administration of ethanolic extract of leaves of a mangrove species plant,* Ceriops decandra*, at 120 mg/kg for 30 days to alloxan-induced diabetic rats showed similar results on blood glucose, hemoglobin, acetylated hemoglobin, and liver glycogen similar to the standard antidiabetic drug, glibenclamide [55]. Antidiabetic effect in alloxan-induced diabetic rats has also been observed with extract of leaf powder of the mangrove species,* Rhizophora mucronata*,* Rhizophora apiculata*, and* Rhizophora annamalayana* [56]. A leaf suspension of the black mangrove species,* Aegiceras corniculatum*, also reportedly gave antidiabetic effects following administration to alloxan-induced diabetic rats, which effects included decreases in blood glucose, glycosylated hemoglobin, decrease in activities of the enzymes, glucose-6 phosphatase and fructose 1,6-bisphosphatase, and increased activity of liver hexokinase [57].

### 5.2. Antimicrobial Activity

*Heritiera fomes *leaf (at doses of 250 *μ*g/disc and 500 *μ*g/disc) exhibited potent antimicrobial activity with the zones of inhibition against tested gram-positive and gram-negative pathogens covering from 3.92 to 7.63 mm; and 7.86 to 13.45 mm, respectively [25].

The bark extracts of* H. fomes* reported significant antibacterial activities against* P. aeruginosa, S. aureus, K. rhizophila,* and* B. subtilis* [12].

*In vitro*, antibacterial comparative study between the pneumatophores of* Xylocarpus moluccensis* and* H. fomes* demonstrated similar antibacterial profiles in the most cases presenting the zone of inhibitions >10 mm. Extracts of pneumatophores of* H. fomes *exhibited a potent zone of inhibition against* Enterobacter aerogenes.* The diameter of zones of inhibition encompassed between 19 and 21 mm. The MIC (minimum inhibitory concentration) of* H. fomes* extract was evaluated by broth dilution method and showed significant minimum inhibitory concentration (MIC = 400 and 500 *μ*g/mL) against* Shigella boydii* and* Shigella sonnei*, respectively [54].

### 5.3. Antioxidant Activity and Various Related Effects

Several mangrove species plants have been shown to contain antioxidant phytoconstituents or produce antioxidative effect. Leaves extract of* Heritiera fomes* was accessed for both quantitative and qualitative antioxidant activity. Quantitative assay technique was accomplished through DPPH assay (hydrogen donation assay) and qualitative assay was carried out through thin layer chromatographic technique followed by DPPH spray. Leaves extract exhibited significant antioxidant activity with the IC_50_ (50 percent inhibitory concentration) value of 26.30 *μ*g/mL [25].

Bark extracts of* Heritiera fomes* showed potent antioxidant activity with 50% inhibitory concentration (IC_50_) value of 22 *μ*g/mL and effective concentration (EC_50_) value of 19.4 *μ*g/mL, respectively [12].

Aqueous extract of bark of* Rhizophora mangle* has been shown to show a protective action against diclofenac-induced gastric ulcer in rats. The protective effect was attributed to antioxidant activity of the extract, since administration of the extract led to marked increases in glutathione peroxidase and superoxide dismutase activity and inhibition of lipid peroxidation [58]. Antiulcer activity with bark extract of this plant has also been observed in acetic acid-induced gastric ulcer model in rodents [59]. Three dammarane triterpenes, bruguierins A–C, have been isolated from* Bruguiera gymnorrhiza* with antioxidative capacity. Additionally, bruguierin A inhibited phorbol ester-induced nuclear factor-*κ*B activation and selectively inhibited cyclooxygenase-2 activity [60]. Butanolic fraction of bark extract of* Rhizophora mangle* has been shown to give protective action in rats against gastric damage induced by absolute ethanol and ischemia-reperfusion. The protective action has been attributed to polyphenols present in the fraction and their antioxidative effects [61].

Sulfated polysaccharides present in* Rhizophora apiculata* bark extract has been shown to play a protective role through their free radical scavenging properties against naphthalene-induced mitochondrial dysfunction [62]. Iridoid glucosides and flavones have been isolated from aerial parts of* Avicennia marina* with *α*,*α*-diphenyl-*β*-picrylhydrazyl (DPPH) radical scavenging activities [63]. A new acetylated flavanol, 3,7-*O*-diacetyl (-)-epicatechin, and other flavanol derivatives have been reported from stems and twigs of* Rhizophora stylosa*. The compounds showed DPPH radical scavenging activities [64]. Flavan-3-ol glycosides and flavan-3-ols with DPPH scavenging activity have also been described from stems of this plant [65]. The total phenolic content and antioxidative activity of methanolic extract of leaves of* Rhizophora mucronata* have been reported to be very high [66].

Condensed tannins showing antioxidant activity have been isolated from the mangrove species,* Kandelia candel* and* Rhizophora mangle* [67]. A 70% acetone extract from the hypocotyls of* K. candel* and its various fractions of petroleum ether, ethyl acetate, and water also reportedly showed DPPH free radical scavenging activity. Further analysis showed that phenolic compounds present within the extract and its various fractions were responsible for the observed activity and these compounds contained a large number of procyanidins and a small amount of prodelphinidins, and epicatechin was the main extension unit [68]. Antioxidant and hepatoprotective effects have been observed with leaf extract of* Lumnitzera racemosa*, which activities have been attributed to phenolic groups, terpenoids and alkaloids in the extract [69].

Antioxidant activities as measured by DPPH radical scavenging and other assays have been reported for bark extract of* Bruguiera cylindrica* and* Ceriops decandra* [70]. Antioxidant activities like restoring the levels of reduced glutathione (GSH) and the antioxidant enzymes superoxide dismutase (SOD), catalase (CAT), and glutathione peroxidase (GPX), along with significant inhibition of both lipid peroxidation and myeloperoxidase (MPO) activity, have been seen with administration of methanolic extract of* Acanthus ilicifolius* leaf extract in different models of gastric ulceration. It has been hypothesized that gastroprotective action of the extract was due to its antioxidant properties [71]. Jacaranone analogs, marinoids F-1 (1–4) have been reported from fruits of* Avicennia marina*, with one of the isolates (4) showing good antioxidant activity [72].

### 5.4. Antinociceptive and Anti-Inflammatory Activity

Oral administration of* Heritiera fomes *bark extracts (at doses of 100, 250, and 500 mg/kg) significantly reduced the number of inhibition of acetic acid-induced writhings in mice by 8.5, 26.4, and 43.4%, respectively, while aspirin (250 mg/kg) used as reference drug showed 20.9% writhing inhibition [11].

Leaf extracts of* Heritiera fomes* (at the doses of 250 and 500 mg/kg body wt) significantly (*P* < 0.001) showed 34.83% and 59.20% writhing inhibition, respectively, while diclofenac sodium (25 mg/kg) used as reference drug showed 70.65% writhing inhibition. In hot plate test method, leaf extracts (500 mg/kg) exhibited highest nociceptive inhibition and took more time for reaction that was comparable to morphine sulfate (standard drug) at the dose of 5 mg/kg [25].

Lupeol has been reported from the mangrove species,* Sonneratia apetala* [73]. Antinociceptive property has been described for this compound [74]. Betulinic acid has been reported from both* Sonneratia apetala* [73] and* Ceriops tagal* [75]. Anti-inflammatory, analgesic, and antipyrexic effects of this compound isolated from* Tetracera potatoria* have been reported [76]. Inhibition of cyclooxygenase-2 (COX-2) activity (which can result in analgesic and anti-inflammatory effects through inhibition of prostaglandin biosynthesis) has been reported for bruguierin A, isolated from* Bruguiera gymnorrhiza* [60]. The aqueous extract and polyphenolic fractions of* Rhizophora mangle* has also been shown to inhibit COX-2 activity [77].

Diterpenoids isolated from stems and twigs of* Excoecaria agallocha* showed anti-inflammatory potency to suppress expression of nuclear factor-*κ*B and AP-1 targeted genes including tumor necrosis factor-*α* (TNF-*α*) and interleukin-6 (IL-6) induced by lipopolysaccharide (LPS) in mouse macrophages Raw 264.7 cells [78]. The anti-inflammatory effect of* Cerbera manghas* methanol extract has been shown to be due to presence of kaempferol in the extract [79].

### 5.5. Anticancer Activity

Phytochemical assessment of the leaves extract of* Heritiera fomes* reported the existence of saponins, reducing sugars, alkaloids, glycosides, flavonoids, tannins, steroids, and gums [25]. In addition, proanthocyanidins (bioactive compounds, present in various medicinal plants including* Heritiera fomes*) reportedly possess antiviral, antibacterial, enzyme inhibiting, antioxidant, and anticarcinogen properties [53, 80, 81]. Both leaves and stems extract of* Heritiera fomes* demonstrated anticancer properties against B16 mouse melanoma (*in vitro* system) with 40% inhibition and EAC (Ehrlich Ascites Carcinoma) in Swiss albino mice (*in vivo* system) [13].

Methanolic extract of* Rhizophora apiculata* has been evaluated for its anti-inflammatory and antitumor activity against B16F10 melanoma cells in BALB/c mice. Administration of extract led to inhibition of solid tumor development in mice. Extract treatment significantly reduced tumor cell GSH, *γ*-glutamyl transpeptidase (GGT) and nitric oxide (NO) levels in the tumor bearing animals. Analysis of the extract revealed presence of 4-pyrrolidinyl, pyrazole, and ketone derivatives [82].

Several other mangrove species have been reported with anticancer potential. Antitumor activity of 3-chlorodeoxylapachol, a naphthoquinone obtained from* Avicennia germinans* has been demonstrated [83]. Cytotoxicity of limonoids isolated from seeds of* Xylocarpus granatum* against P-388 and A-549 tumor cell lines has been reported [84]. Gedunin is a limonoid isolated from the same plant demonstrated inhibition of growth of CaCo-2 colon cancer cell line* in vitro* [85]. Another limonoid, xylomexicanin A, isolated from the same plant, demonstrated antiproliferative activity against human breast carcinoma, KT cells [86]. Black tea extract of* Ceriops decandra* has been shown to prevent dimethyl benz[a]anthracene (DMBA)-induced buccal pouch carcinogenesis in hamsters [87].

Patriscabratine and tetracosane, isolated from the Bangladesh mangrove fern,* Acrostichum aureum*, reportedly showed moderately cytotoxic activity against AGS, MDA-MB-231 and MCF-7 cells, and AGS, MDA-MB-231, HT-29, and NIH 3T3 cells, respectively [88]. The sesquiterpene, (2R, 3S)-sulfated pterosin C, isolated from the same fern demonstrated an apoptotic effect on AGS cells within 24 hours of treatment [89]. Methanolic extract of* Rhizophora apiculata* reportedly inhibited B16F-10 melanoma induced lung metastasis in C57BL/6 mice [90]. Taken together, the various reports indicate that mangrove species plants may be good sources of potential anticancer drugs.

## 6. Future Prospects for Medicinal Discoveries

This review revealed that* H. fomes* possesses significant pharmacological potential and chemical constituents and rural people living beside the Sundarbans use this plant to treat a wide spectrum of human ailments. It is necessary to accumulate this indigenous knowledge by proper documentation and preserve it for future research. In spite of having promising biological and pharmacological potentials, there are only few reports are available on this plant. More research is required on the phytochemical constituents of this plant. This review will help the future researchers in discovering new therapeutic agents as the plant possesses propitious biological and pharmacological potentials.

Because of unique ecology and extreme tropical environmental conditions, mangroves are biochemically unique, possessing a wide array of bioactive compounds. They are the prime source of novel compounds having multifarious pharmacological activities. Compounds that are isolated from the mangrove species has the potential to act as lead compounds for drug discovery. In spite of having enormous ethnomedicinal and folklore applications, these plants remain unexplored.

Mangrove plants contain secondary metabolites like alkaloids, phenolics, steroids, and terpenoids with pharmacological, ecological, and toxicological importance, for instance, phytoconstituents (alkaloids, flavonoids, terpenoids, phenolics and saponins of two mangrove species,* Avicennia marina,* and* Avicennia officinalis*, have reported antimicrobial properties) [91]. An* in vitro* investigation demonstrated the presence of saponins, flavonoids, phenols, polyphenols, tannins, and reducing sugars in* Heritiera fomes*. Some of these compounds like flavonoids, phenols, and polyphenols can act as antioxidants and scavenge free radicals [92, 93]. Some highly reactive free radicals like nitrogen and oxygen species (RNS, ROS) have the potential to cause oxidative injury to DNA, proteins, lipids, and resulting in various diseases [94]. Antioxidants give protection to cells against oxidative damage [95]. Proanthocyanidins and its metabolites and hydroxylated phenolic acids have high antioxidant activity [96, 97]. An investigation acknowledged that proanthocyanidin-rich products and proanthocyanidins possess substantial RNS/ROS scavenging activity [53]. Due to the presence of high amount of procyanidins, the plant (*Heritiera fomes*) can act both as radical scavenger and 15-LO inhibitor. A derivative of procyanidin B2 reportedly acted against human promyelocytic leukemia cells (HL-60) and melanoma cell line [98]. Mixture of procyanidins reportedly induced cytotoxic activity in human squamous cell carcinoma [99]. From the above discussion it is clear that the plant may serve as a good source of anticancer phytomedicines.

Since the plant possesses significant biological and pharmacological activity it should be explored for its medicinal value at molecular level by using various modern scientific techniques. The plant has a wide array of biological and pharmacological potentials and many of the isolated compounds and synthetic analogues of* Heritiera fomes* merit further research.

## 7. Conclusion 

Several* in vitro* studies established that the plant possesses significant antioxidant, antinociceptive, antimicrobial, antidiabetic, and anticancer activities. It is very necessary to isolate the bioactive compounds responsible for these activities. In this regard, systematic approaches such as bioassay-guided fractionation can be considered. The present study should serve as a basis and an important tool for future chemical screening and biological assays and should open new perspectives for new drug discovery.

## Figures and Tables

**Table 1:** Different parts of *H*. *fomes* with mode of preparation and administration, medicinal uses/feature, and disease category.

Part(s) used	Mode of preparation	Medicinal use(s) in common diseases and features	Disease category	References
Leaves and Seeds	Decoction	Diarrhea, dysentery, colic, acidity, indigestion, constipation, stomachache, bloating, and lack of appetite	Gastrointestinal disorders	[8, 9, 26]

Wood	Powder	Piles	Rectal diseases	[27]

Stem bark	paste	Eczema, abscess, boils, acne, infections, scabies, itch, dermatitis, rash, sores, scar, and warts	Skin diseases	[8, 26, 28]

Bark	Hot decoction	Diabetes and goiter	Diabetes	[11, 13]

Twig	Toothbrush	Toothache and oral infection	N/A	[29]

**Table 2:** Phytochemical constituents obtained from* H. fomes*.

Plant Parts	Phytochemical constituents reported	References
Leaves	0.25% chlorophyll a	[30]
	0.09% chlorophyll b	
	0.11% carotenoids	
	39.45% polyphenols	
	21.12% tannins	
	29.22% proteins	

Phytochemical exploration of leaf extract	Reducing sugars, saponins, alkaloids, glycosides, tannins, steroids, flavonoids, and gums	[25]

Bark	7–36% tannin, high content of proanthocyanidins	[31, 32]

Stem bark	Trimeric, pentameric and hexameric procyanidins	[12]

NMR spectroscopy of CHCl_3_ extract	*β*-Sitosterol, stigmasterol, and stigmast-4-en-3-one	[12]

**Table 3:** Observed pharmacological activity of *H. fomes* in various test methods with different solvent extractions.

Solvent extraction and plant part(s) tested	Observed activity	Test method	References
EtOH extracts of leaves.	Antinociceptive	Hot plate, acetic acid-induced writhings in mice	[25]
	Antioxidant	DPPH radical scavenging assay	
	Antimicrobial	Disk diffusion assay	

80% EtOH crude, CHCl_l3_, EtOAc, BuOH, aqueous residue extracts of stem bark, and pure compounds	Antioxidant	15-Lipoxygenase inhibition, total phenolic content, and DPPH radical scavenging assay	[12]
80% EtOH crude, CHCl_3_, EtOAc, BuOH, aqueous residue, precipitate extracts of stem bark, and negative control (acetone, MeOH)	Antimicrobial	Agar disc diffusion method	

MeOH extract of bark	Antihyperglycemic	Lowering serum glucose level in hyperglycemic mice following of glucose loading	[11]
	Antinociceptive	Acetic-acid-induced writhings in mice	

MeOH extract of both leaf and stem powder	Anticancer activity	In vitro cell viability and In vivo screening assay against B16 mouse melanoma and EAC (ehrlich ascites carcinoma) in mice model	[13]
	chromatography characterization	TLC (qualitative and quantitative DPPH assay), HPLC, 1H NMR, FTIR spectral analysis, and bioautography screening	

EtOH extracts of pneumatophores	Comparative antibacterial activity	Minimum Inhibitory Concentration (MIC) method	[54]

## Glossary

CAT: Catalase
COX-2: Cyclooxygenase-2
DMBA: Dimethyl benz[a]anthracene
DPPH: *α*,*α*-Diphenyl-*β*-picrylhydrazyl
EAC: Ehrlich ascites carcinoma
GGT: *γ*-Glutamyl transpeptidase
GSH: Reduced glutathione
GPX: Glutathione peroxidase
HL-60: Human promyelocytic leukemia cells
IL-6: Interleukin-6
IUCN: International Union for Conservation of Nature
15-LO: 15-Lipoxygenase
LPS: Lipopolysaccharide
MPO: Myeloperoxidase
NMR: Nuclear magnetic resonance
NO: Nitric oxide
RNS: Reactive nitrogen species
ROS: Reactive oxygen species
SOD: Superoxide dismutase
TAN: Titratable acid number
THPs: Traditional health practitioners
TNF-*α*: Tumor necrosis factor-*α*.
